# *COVID Alert*: Factors Influencing the Adoption of Exposure Notification Apps Among Canadian Residents

**DOI:** 10.3389/fdgth.2022.842661

**Published:** 2022-03-11

**Authors:** Kiemute Oyibo, Plinio Pelegrini Morita

**Affiliations:** School of Public Health Sciences, University of Waterloo, Waterloo, ON, Canada

**Keywords:** technology acceptance model, contact tracing app, exposure notification app, persuasive design, adoption, COVID-19, COVID Alert

## Abstract

The continued emergence of new variants of COVID-19 such as the Delta and Omicron variants, which can cause breakthrough infections, indicates that contact tracing and exposure notification apps (ENAs) will continue to be useful for the long haul. However, there is limited work to uncover the strongest factors that influence their adoption. Using Canada's “COVID Alert” as a case study, we conducted an empirical, technology-acceptance study to investigate the key factors that account for users' intention to use ENAs and the moderating effect of important human and design factors. Our path model analysis shows that four factors significantly influence the adoption of COVID Alert among Canadian residents: perceived risk, perceived usefulness, perceived trust, and perceived compatibility. The overall model explains over 60% of intention to use, with type of design, use case (functional interface), and adoption status moderating the strength of the relationships between the four factors and intention to use. We discuss these findings and make recommendations for the design of future ENAs.

## 1. Introduction

Contact tracing apps (CTAs) have become a new buzzword in the public health literature since the outbreak of the COVID-19 coronavirus in the early part of 2020 (1). CTA is a mobile-technology-based system for logging, tracking and contacting people who may have come into close contact with a person infected by the coronavirus. Since the inception of the COVID-19 pandemic, CTAs, particularly exposure notification apps (ENAs), have been deployed worldwide by national governments to curb the spread of the coronavirus. In general, there are two main types of CTAs: Bluetooth and global positioning system (GPS) enabled. The main difference between Bluetooth and GPS-based contact tracing is that, in the former, users' locations are not tracked, but, in the latter, they are tracked, thereby raising privacy concerns in many countries in which the GPS-enabled CTAs are in use. In most countries, Bluetooth technology is used to determine and track direct face-to-face interactions by collecting the Bluetooth IDs of both mobile phones that come in close contact. For example, Singapore was the first country to implement a Bluetooth-enabled CTA called “TraceTogether” released on 20 March 2020 (2). The app allows the Singaporean government to identify possible infected people and quarantine them (3). Moreover, in some other countries, GPS technology is being used to track infected people and their contacts. For example, China and Israel use GPS-enabled CTAs to acquire the contact details of infected people and their status. The continued emergence of new variants of the COVID-19 virus, which are resistant to the currently developed vaccines being deployed worldwide, suggests that CTAs will remain relevant for a very long time in the fight against the spread of the virus. However, various concerns surrounding privacy have been raised by stakeholders and the general public, especially with regard to how GPS-enabled CTAs may be used post-COVID, for example, to trace the movements of individuals and their contacts (4, 5).

Due to privacy concerns and data protection laws worldwide, Apple and Google developed a Bluetooth-enabled, privacy-preserving system called “Google/Apple Exposure Notification (GAEN) System” (6), which allows developers to implement ENAs in a privacy-preserving and data-decentralized way. The GAEN system, through application program interfaces (APIs), allows government-sponsored developers to roll out ENAs quickly. However, there is poor a uptake of the current ENAs on the market due to a number of application design and human-factor issues. According to Kukuk (2), “*CTAs might turn out to be a failure, as [his] research finds a low intention to use such apps*” (p. 1). Hence, it becomes pertinent for researchers to understand the key factors that determine the adoption of ENAs currently published in the Google and Apple stores. This will help designers focus on the important factors to increase the adoption and effectiveness of ENAs. Although a number of studies have been carried out on technology acceptance of CTAs, most of the studies are among non-Canadian residents, the result of which may not generalize to the Canadian population. In particular, most of the existing studies did not investigate the moderating effect of important demographic factors such as adoption status. Hence, to uncover the key factors that predict the adoption of CTAs among Canadian residents, we used the Government of Canada's COVID Alert app as a case study. We conducted an online survey to investigate the technology acceptance model (TAM) for the COVID Alert app among Canadian residents and the moderating effect of type of design, use case (functional interface), and adoption status. In this paper, we present the results of our findings based on path models and provide design recommendations to improve future iterations of ENAs.

## 2. Background

In this section, we provide an overview of the TAM and ENA. The overview of the TAM provides a theoretical basis for the current work, while the overview of ENA provides an insight into how it functions.

### 2.1. Technology Acceptance Model

The TAM was proposed by Davis (7) as a conceptual framework for understanding the key factors that explain the acceptance of a new information technology. The TAM has evolved over the years into various variants. Currently, one of the most commonly adopted variants of TAM is the Unified Theory of Acceptance and Use of Technology (UTAUT) model, which was developed by Vankatesh et al. (8) from a systematic review of the existing literature. The UTAUT is based on existing models, including Theory of Reasoned Action, Theory of Planned Behavior, the original TAM, Motivational Model, Model of Personal Computer Use, Diffusion of Innovations and Social Cognitive Theory. The UTAUT provides a unified and compact theory of acceptance of information technology based on four main driver constructs (Performance Expectancy, Effort Expectancy, Social Influence, and Facilitating Conditions), which have the potential to predict Behavioral Intentions. Overtime, the UTAUT model has been extended to other variants, which include other important predictors such as Hedonic Motivation (Perceived Enjoyment), Privacy Concern, Perceived Trust, Perceived Risk, Perceived Persuasiveness, etc. (9). In the context of CTAs, Table 1 shows the constructs in our extended UTAUT model and their definitions.

**Table 1:** UTAUT constructs and their definitions.

**Construct**	**Definition**
Perceived Usefulness	The degree to which users believe that an ENA will accomplish its purpose (10).
Perceived Ease of Use	The degree to which users believe that the usage of an ENA will be free of efforts (10).
Privacy Concern	The concern about the loss of privacy due to the use of an ENA and disclosure of user data (11).
Perceived Trust	The belief that an ENA is credible and trustworthy.
Perceived Risk	The concern about whether an ENA will violate its privacy and confidentiality norms (12).
Perceived Enjoyment	The fun or pleasure users derive from using an ENA (13).
Perceived Compatibility	The degree to which an ENA is perceived as being consistent with past user experience (14).
Intention to Use	The plan or intention to use (or continue using) an ENA to curb the spread of the coronavirus.

*In other studies, Perceived Usefulness = Performance Expectancy, Perceived Ease of Use = Effort Expectancy*.

### 2.2. Exposure Notification App

**Figure 1:**
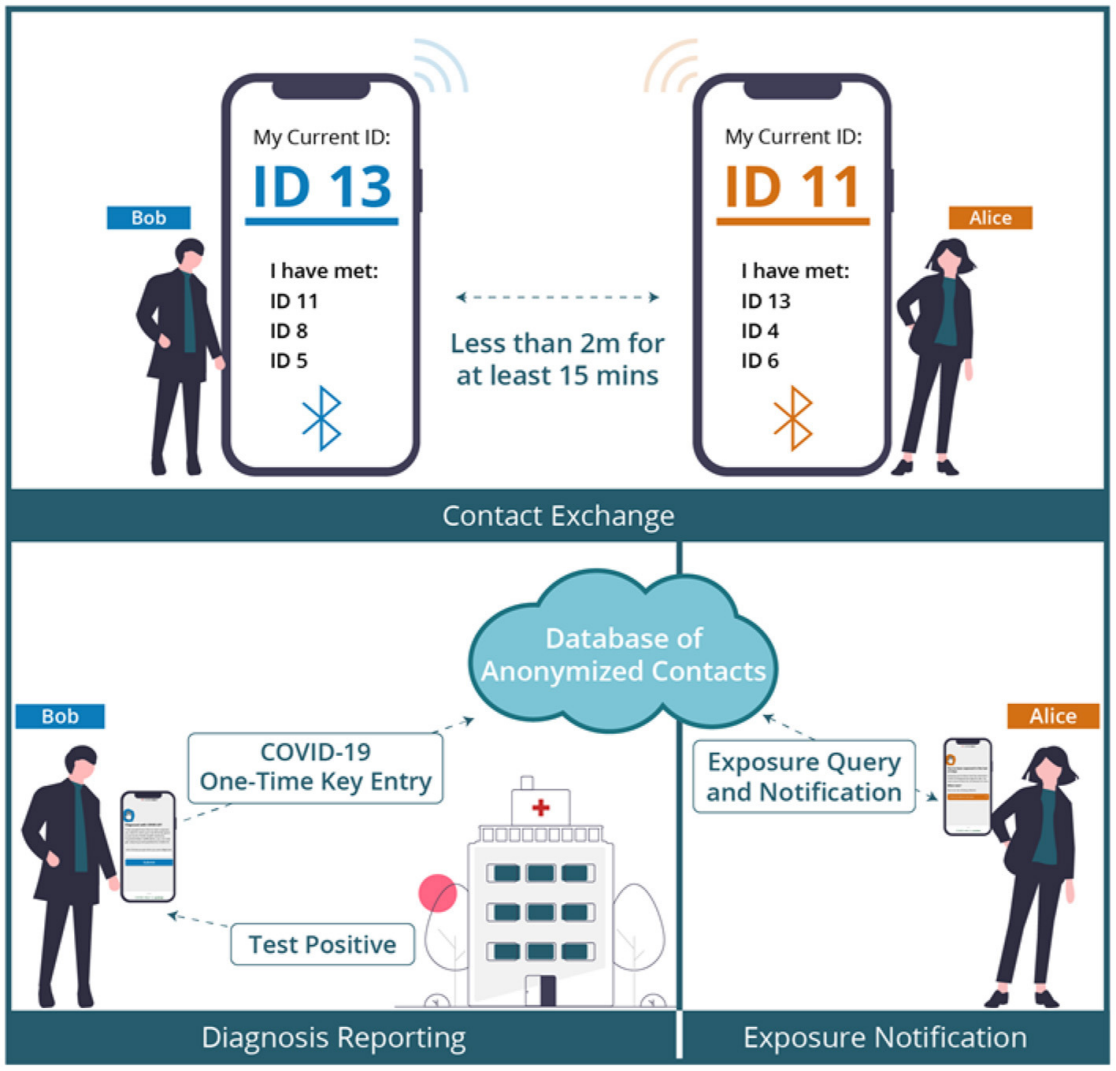
COVID-19 contact tracing and exposure notification process (15).

The ENA is a mobile app designed to alert users that may have come in close contact with someone infected with COVID-19. Countries such as Canada (COVID Alert), Australia (COVID Safe), Singapore (Trace Together), South Africa (COVI-ID), and United Kingdom (NHS COVID-19 App) developed their respective national ENAs to curb the spread of the COVID-19 virus. The overall functionality of the ENAs can be illustrated in Figure 1 using two hypothetical contacts: Alice and Bob. If Alice comes in close contact with Bob (i.e., within 2-m distance) for 15 min or more, both contacts exchange a dynamic randomly generated ID. At a later time or date, if Alice tests positive and uploads her one-time key given to her by the public health authority, Bob will be contacted *via* the exposure notification interface and advised on what to do, e.g., self-isolate for at least 14 days or go test for COVID-19 if having symptoms.

## 3. Related Work

A number of studies have been conducted to investigate the factors that explain the acceptance/adoption of CTAs. Velicia-Martin et al. (16) conducted a TAM study by recruiting participants on social media and by email. In their model based on the combination of TAM and Health Belief Model (HBM) constructs, they found that Attitude (toward using technology), Perceived Usefulness, and Perceived Ease of Use had a strong effect on users' Intention to Use CTAs, while Perceived Trust and Perceived Risk (to be infected with COVID-19) had a weak effect. It turned out that Privacy Concern had no significant effect on CTA adoption. However, the study was based on a description of CTA; it was carried out in the first half of 2020 when a lot of people were yet to be familiar with CTAs. Trang (17) conducted a study to investigate the Intention to Install CTAs among the German populations. The author found that Convenience Design, Privacy Design, Coronavirus Anxiety, Social Benefit (compared with Self-Benefit), and information Technology (IT) Self-Efficacy were facilitators of CTA adoption, while Privacy concern was a barrier. However, just like Velicia-Martin et al. (16), the study was conducted in the early first half of 2020 when very few people were familiar with CTAs, let alone not considering key UTAUT constructs such as Perceived Ease of Use and Perceived Trust. Walrave et al. (18) investigated the predictors of Adoption Intention of CTA among Belgium residents using the extended UTAUT model. They found that Performance Expectancy (aka Perceived Usefulness) is the most important (positive) predictor of Adoption Intention, followed by Facilitating Conditions and Social Influence. Moreover, Privacy Concerns negatively influenced Adoption Intention, while Effort Expectancy (aka Perceived Usefulness) was not related. Kukuk (2) conducted a study among German, Dutch, British, American, and other national populations to investigate the adoption of CTAs using the UTAUT as an analytical model. The author found that Performance Expectancy and Perceived Credibility had a significant effect on the Intention to Use CTAs. However, these three constructs, which are important to adoption, were perceived low by the study participants, which is an indication that the participants were unwilling to accept and use the CTAs in general. The main limitation of this study as well as Walrave et al.'s is that the survey questions were based on CTAs in general and not a specific national app, which the respondents had knowledge of, must have used, were familiar with, or were presented with in the survey to visualize its capabilities. These factors have the potential of influencing the relationships between the predicting constructs and Intention to Use in the UTAUT model.

Moreover, Abuhammad et al. (19) investigated the acceptance of CTAs and the ethical issues associated with them among Jordanians. They found that income and living area were predictors of acceptability and use of contact tracing technology, with participants being particularly concerned with ethical issues relating to privacy, voluntariness, and beneficence of the data. However, this study was not carried out in the context of TAM, thereby not taking into consideration important UTAUT-based factors such as Performance Expectancy and Facilitation Condition, which may be significant determinants of CTA adoption. Utz (20) conducted an online study in Germany, United States, and China to investigate the acceptance of CTAs. They found that acceptance was highest in China and lowest in the United States. Moreover, they found that Chinese respondents were less concerned about privacy. For example, while Chinese respondents favored the collection of personalized data, German and American respondents preferred anonymity. However, the study was based on a vignette design of hypothetical CTAs inspired by existing apps worldwide. Secondly, the study was neither based on the TAM/UTAUT constructs nor Canadian population, unlike our current study. Finally, Bohm (21) carried out a mixed study of the German population to analyze the barriers to the adoption of the German CTA and the effect of a video intervention. The authors collected data based on TAM measures before and after participants watched the video. The authors found that the video intervention did not significantly increase participants' Behavioral Intention. However, they found that the average scores of Behavioral Intention, Perceived Ease of Use, and Perceived Usefulness, increased significantly with high and medium effect sizes. Moreover, their qualitative data analysis revealed that Perceived Risk/Costs (high) and Perceived Personal Benefits (low) were the main barriers to CTA adoption. However, the study did not investigate important UTAUT constructs such as Privacy Concern, Perceived Risk, and Perceived Trust, which our current study considered.

To bridge the existing gaps in the literature, the objective of our current paper is to focus on: (1) an actual national app currently be used (Government of Canada's COVID Alert), (2) a wide range of UTAUT constructs (including Perceived Enjoyment, a hedonic construct), (3) the moderating effect of type of design (persuasive vs. control), use case (exposure monitoring vs. diagnosis reporting), and adoption status (CTA adopters vs. non-adopters), and (4) the Canadian population yet to be investigated.

## 4. Method

This section covers our research question, research design, research model and hypotheses, measurement instruments and participants' demographics.

### 4.1. Research Questions

Based on our research objective on technology acceptance, we aim to answer the following research questions:

(1) What factors are the strongest determinants of the adoption of ENAs among the Canadian population?
(2) Are the relationships between the determinants and intention to use ENAs moderated by the type of design, use case, and adoption status?

### 4.2. Research Design

**Figure 2:**
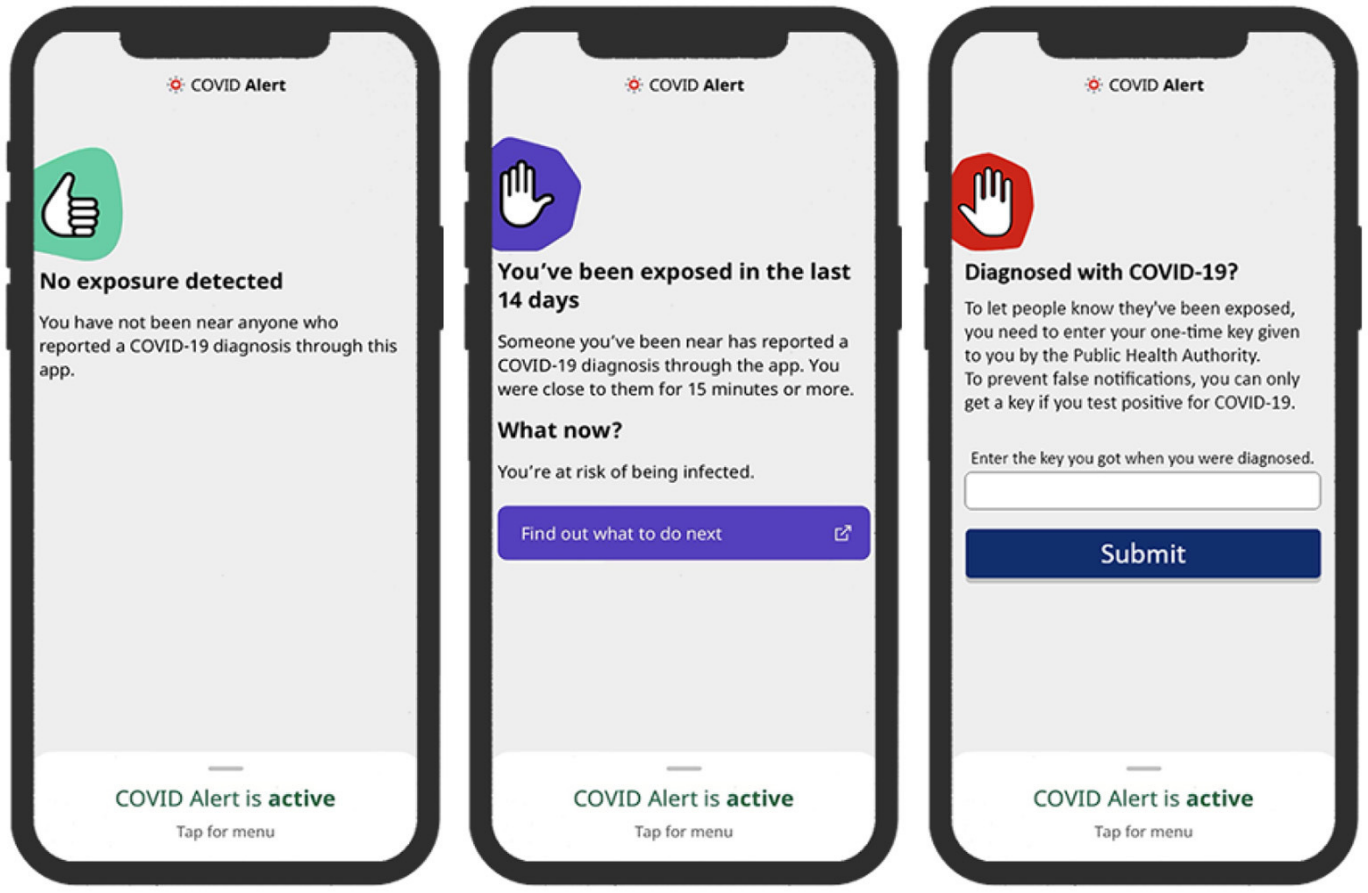
Control design of COVID Alert (**left**: no-exposure interface, **middle**: exposure interface, **right**: diagnosis-report interface).

**Figure 3:**
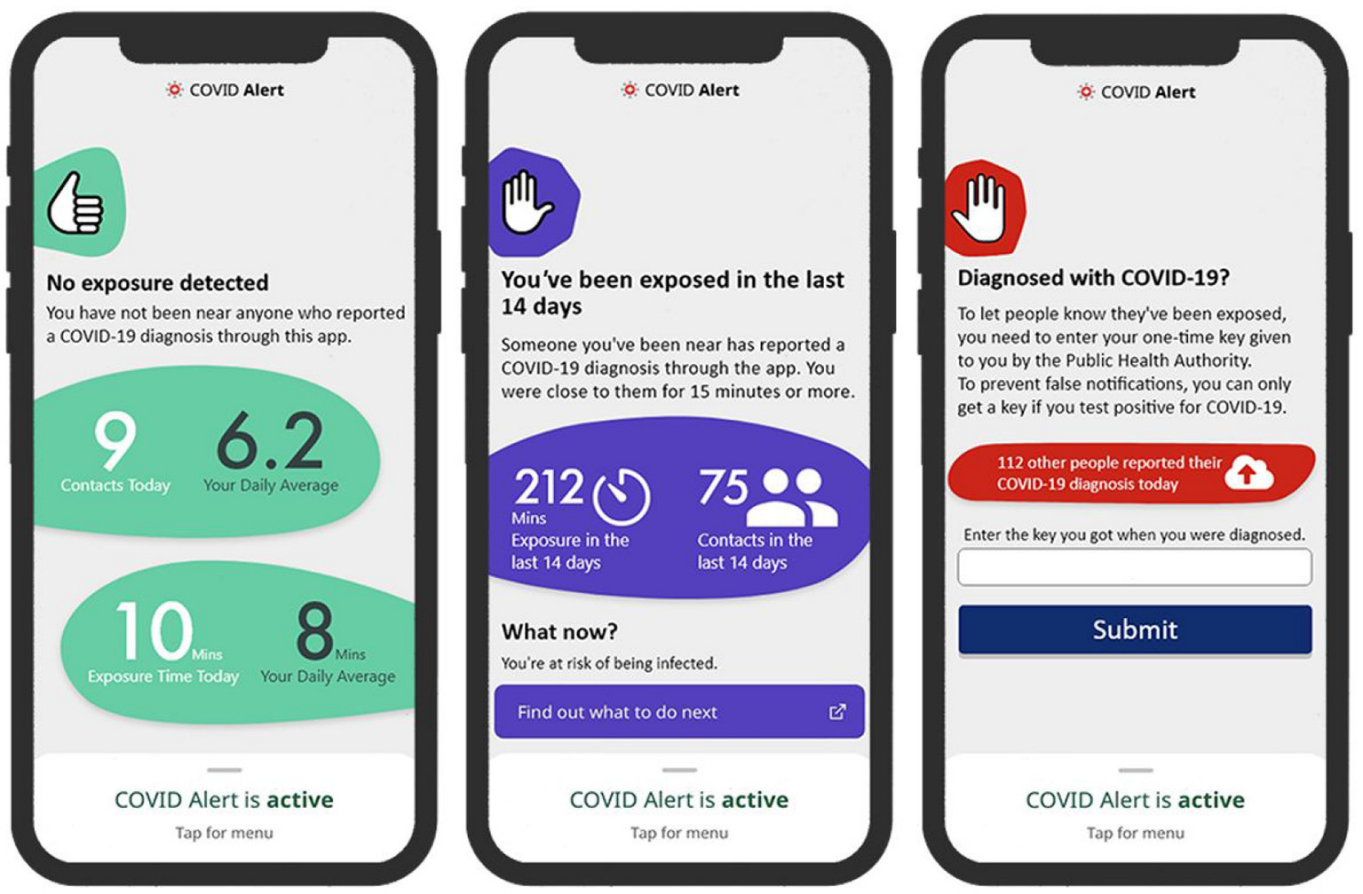
Persuasive design of COVID Alert (**left**: no-exposure interface, **middle**: exposure interface, **right**: diagnosis-report interface).

**Figure 4:**
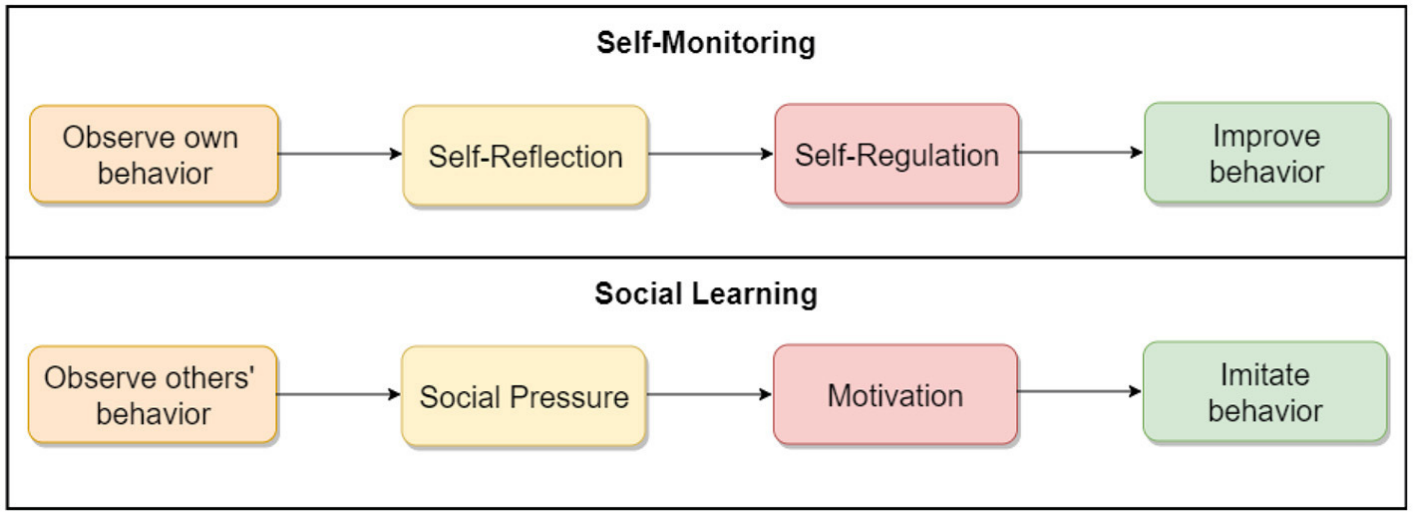
The operational mechanism of self-monitoring and social learning (23, 24).

To address our research questions, we designed two sets of three key interfaces in the COVID Alert app. The two sets represent two different types of app design: control design (Figure 2) and persuasive design (Figure 3). The three key interfaces represent the three use cases of an ENA: no-exposure status, exposure status, and diagnosis report. Specifically, the control designs represent the actual COVID Alert app, with slight adaptations to suit the purpose of our study. For example, in the diagnosis-report interface, the red hand icon was not in the original version. However, for consistency across all three interfaces, we decided to include the red hand icon in the third interface. All of the six interfaces were randomly assigned to six different groups in our study. Regarding the designs shown in Figure 3, the first two user interfaces implemented self-monitoring, while the third user interface implemented social learning. Both persuasive strategies were drawn from the Persuasive System Design Model: a framework proposed by Oinas-Kukkonen and Harjumaa (22) for designing, implementing, and evaluating persuasive systems. Figure 4 shows the operational mechanism of self-monitoring and social learning. Self-monitoring is regarded as one of the cornerstones of persuasive technology, which enables users to track their own behavior and make improvement (25). As shown in Figure 4, through self-monitoring, users are able to reflect on their performance. If they are not satisfied with their performance, they make improvement through self-regulation. On the other hand, social learning is the observation of others' behavior and imitating it. As shown in Figure 4, when users observe the behavior of others, they are motivated through social pressure to imitate the observed behavior if it is beneficial to them, others, or society in general. According to (26), social learning has the potential to encourage, challenge, and motivate the user to engage in the observed behavior.

#### 4.2.1. No Exposure Status UI

This interface, which we call no-exposure interface, for short, contains the no-exposure-to-COVID-19 status information, “*You have not been near anyone who reported a COVID-19 diagnosis through this app*.” In addition, the persuasive version implements a self-monitoring persuasive feature, which allows the user to track the number of their daily contacts and exposure time. This feature, through self-regulation, has the potential of increasing the user's commitment and focus on achieving the target behaviors, e.g., staying at home and maintaining social distancing when in public places.

#### 4.2.2. Exposure Status UI

This interface, which we call exposure interface, for short, contains the exposure-to-COVID-19 status information, “*Someone you've been near has reported a COVID-19 diagnosis through the app. You were close to them for 15 minutes or more*.” In addition, the persuasive version in Figure 3 implements a self-monitoring persuasive feature, which shows the overall exposure level of the user in the last 14 days.

#### 4.2.3. Diagnosis Report UI

This interface provides an interface to report a COVID-19 diagnosis. It prompts the user with the information, “*Enter the key you got when you were diagnosed. To prevent false notifications, you can only get a key if you test positive for COVID-19*.” In addition, the persuasive version in Figure 3 implements a social-learning persuasive feature, which raises awareness about the number of people that have reported their COVID-19 diagnosis status for the present day. This feature aims to use social pressure to motivate a user who tested positive to report their diagnosis.

### 4.3. Measurement Instruments

The study employed measurement instruments adapted from prior studies in the existing literature to suit the context of ENAs. Table 2 shows the various constructs we measured and their items. Apart from the target construct (intention to use), each of the constructs is composed of 3 items. Their scales range from “Strongly Disagree (1)” to “Strongly Agree (7).” (See Table A1 in Appendix for the description of how the three use cases were administered to participants.) In addition to the measured UTAUT constructs, we asked participants to indicate their CTA adoption status, which includes COVID Alert adopter, CTA/ENA adopter, and non-adopter (Table A1).

**Table 2:** Measurement items for the extended UTAUT constructs.

**Construct**	**Items**
Perceived Usefulness (27)	(1) I find the app to be useful.
	(2) Using the app will increase my awareness about the spread of the coronavirus.
	(3) Using the app will help me in knowing my COVID-19 exposure status.
Perceived Ease of Use (9)	(1) It will be easy for me to become skillful using the app.
	(2) I will find it easy to use the app.
	(3) Learning to operate the app will be easy for me.
Privacy Concern (27)	(1) I feel comfortable giving personal information on this app.^*^
	(2) I feel comfortable using the app.^*^
	(3) The app clearly explains how user information will be used.^*^
Perceived Trust (27)	(1) This app is trustworthy.
	(2) I trust the app keeps my best interests in mind.
	(3) This design of the app meets my expectations.
Perceived Risk (27)	(1) Using the app will involve data privacy risk.
	(2) Using the app will involve data confidentiality risk.
	(3) My overall perception of risk related to using the app is high.
Perceived Enjoyment (9)	(1) Using the app will be fun.
	(2) Using the app will be enjoyable.
	(3) Using the app will be entertaining.
Perceived Compatibility (2)	(1) I have the resources necessary to use the app.
	(2) I have the knowledge necessary to use the app.
	(3) The app is compatible with other technologies I use.
Intention to Use (17)	Overall, if I have the app installed on my mobile phone, I predict I will use or
	continue using it.
Adoption Status	Which of the following best describes you?
	(1) I am currently using the Covid Alert app.
	(2) I am currently using a COVID-19 CTA/ENA other than Covid Alert.
	(3) I am not currently using any COVID-19 CTA/ENA.

* *Item reversed during data analysis*.

### 4.4. Participants

Our study (online questionnaire) was approved by University of Waterloo Research Ethics Committee (ORE #42638). It was then posted on Amazon Mechanical Turk to recruit participants resident in Canada. The recruitment occurred between December 25, 2020 and January 25, 2021. To appreciate participants for their time, each was remunerated with US $2. Table 3 shows the key demographic information of the study participants based n gender, age, education, smartphone usage experience, country of origin, adoption status, and type of design. For example, based on gender, 57.84% of the participants were males, while 38.24% of them were females. Regarding adoption status, the COVID Alert adopters (*n* = 65) and ENA/CTA adopters (*n* = 17) were combined to form the adopter group. Hence, 82 participants belong to the adopter group and 116 participants belong to the non-adopter group.

**Table 3:** Participants' demographics.

**Criterion**	**Subgroup**	**Number**	**Percent**
	Male	118	57.84
Gender	Female	78	38.24
	Others	8	3.92
	18–24	1	0.49
	25–34	41	20.10
Age	35–44	69	33.82
	45–54	53	25.98
	55+	20	9.80
	Unspecified	11	5.39
	Technical/Trade	5	2.45
	High School	39	19.12
	Bachelor	107	52.45
Education	Master	34	16.67
	Doctorate	4	1.96
	PhD	6	2.94
	Other	9	4.41
	1–5	30	14.71
	6–10	95	46.57
Years using smartphone	11-20	64	31.37
	>20	8	3.92
	Unspecified	7	3.43
Country of origin	Canada	158	77.45
	Other	46	22.55
	COVID Alert	65	31.86
Adoption status	Other ENA/CTA	17	8.33
	Non-adopter	116	56.86
	Unspecified	6	2.94
	No-exposure	33	16.18
Control design	Exposure	36	17.65
	Diagnosis-report	32	15.69
	No-exposure	35	17.16
Persuasive design	Exposure	39	19.12
	Diagnosis-report	29	14.22

### 4.5. Research Model and Hypotheses

**Figure 5:**
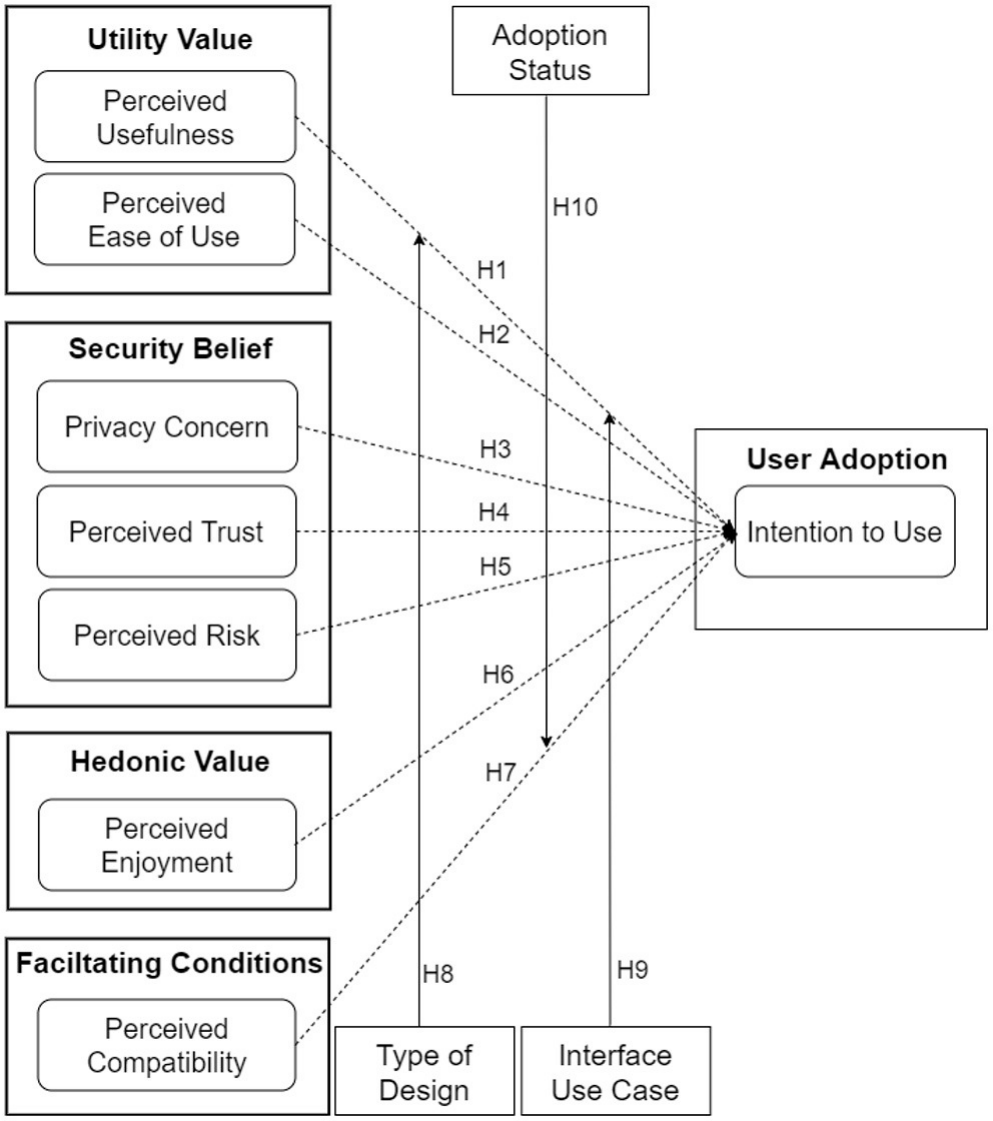
Hypothesized UTAUT model of the adoption of ENAs.

Figure 5 shows our research model of the adoption of an ENA. Adoption is operationalized as intention to use, which plays a mediating role between users' beliefs about an information system and its actual usage (13). Based on Vanketash et al.'s (28) UTAUT, the research model comprises 10 hypotheses divided into two groups: direct and moderating effects. The direct-effect hypotheses comprise five positive relationships (H1, H2, H4, H6, and H7) and two negative relationships (H3 and H5). Moreover, the moderating effect hypotheses are based on the type of design (persuasive vs. control), use case (interface functionality), and adoption status (CTA users vs. non-users).

#### 4.5.1. Utility Value

The first two relationships deal with utility-value constructs [perceived usefulness (H1) and perceived ease of use (H2)]. They are based on prior findings in the literature. Both constructs constitute the two most significant determinants of technology acceptance in the traditional TAM model (2, 10, 29, 30). For example, Van der Heijden (30), in the context of health, found that perceived ease of use has a positive influence on the intention to use health websites. Moreover, in the UTAUT model for CTAs, Kukuk (2) found that perceived usefulness has a positive relationship with intention to use. Based on these prior findings, we hypothesize as follows:

H1. The higher users perceive the design of an ENA to be useful, the higher will be their intention to use it.
H2. The higher users perceive the design of an ENA to be easy to use, the higher will be their intention to use it.

#### 4.5.2. Security Belief

The second set of hypotheses deals with data security concerns such as privacy concern (H3), perceived trust (H4) and perceived risk (H6). They are based on prior findings in the literature. For example, in a study of the acceptance of CTAs, Kukuk (2) found that perceived credibility (which is related to perceived trust) has a positive relationship with intention to use. Moreover, Ernst and Ernst (31), in the physical activity domain, found that perceived privacy risk has a negative influence on users' intention to use smartwatches to support their behavior change. Similarly, in healthcare service delivery, Dhagarra et al. (32) found that privacy concern has a negative impact on the acceptance of a new technology, while perceived trust has a positive influence. Hence, in our current study based on ENAs, we hypothesize as follows:

H3. The higher users' privacy concern about an ENA is, the lower will be their intention to use it.
H4. The higher users perceive the design of an ENA to be trustworthy, the higher will be their intention to use it.
H5. The higher users perceive the design of an ENA to be risky in terms of data privacy and confidentiality, the lower will be their intention to use it.

#### 4.5.3. Hedonic Value

The third set of hypotheses deals with the hedonic value of ENAs (perceived enjoyment). It is composed of one hypothesis (H6), which is based on prior findings in gamified learning environments (GLEs). In this environment, Oluwajana et al. (33) found that the perceived enjoyment or fun created by the expectation of psychological reward can motivate the use of GLEs. Based on this and similar findings in other domains [e.g., (31)], in the context of ENAs, we hypothesize as follows:

H6. The higher users' perceived enjoyment of an ENA is, the higher will be their intention to use it.

#### 4.5.4. Facilitating Conditions

The fourth set of hypotheses (H7) deals with the facilitating-conditions construct (perceived compatibility). Prior research in e-commerce (34) and automation (35) systems found a significant relationship between perceived compatibility and intention to use. In other words, the studies found that the higher users' existing values, needs and experiences are compatible with the new technology, the more likely they are to adopt it. Based on this prior finding, for H7, we hypothesize as follows:

H7. The higher users perceive the design of an ENA to be compatible with the existing applications they have used, the higher will be their intention to use it.

#### 4.5.5. Moderating Effect by Type of Design, Use Case, and Adoption Status

Finally, the fifth set of hypotheses (H8, H9, and H10) deals with the moderating effect of design type, use case, and adoption status. The eighth and ninth hypotheses (H8 and H9) are informed by Sun and Zhang's (36) finding. In their study, the authors found that the nature of a technology and the nature of its functionality (use cases) may influence users' acceptance. Moreover, the tenth hypothesis (H10) is based on the finding that voluntariness, “*the degree to which [the] use of [an] innovation is perceived as being voluntary, or of free will*” (p. 195) (14), moderates the relationships between TAM constructs such as perceived usefulness and intention to use (37). So far, the usage of ENAs/CTAs has been mainly voluntary in many countries, except for few [e.g., India, where it was mandated in certain situations (38)]. Hence, we hypothesize that the adoption status of respondents will moderate some of the relationships in the UTAUT model as follows:

H8. The persuasive design of an ENA will moderate the relationships between some of the hypothesized determinants and the intention to use.
H9. The use case of an ENA will moderate the relationships between some of the hypothesized determinants and the intention to use.
H10. The adoption of an ENA will moderate the relationships between some of the hypothesized determinants and the intention to use.

## 5. Result

This section covers the evaluation of the measurement models, analysis of the structural models, and the multigroup analysis, which allows us to compare the relationships between pairs of groups. The path models were built and analyzed using the “plspm” package in R (39).

### 5.1. Evaluation of Measurement Models

Prior to the path analysis, we evaluated the measurement models to determine their satisfaction of the preconditions necessary for analyzing the structural models. Table 4 shows each of the preconditions, their definitions and the results of the evaluation.

**Table 4:** Results of evaluation of measurement models (39).

**Criterion**	**Definition**	**Evaluation result**
Indicator reliability	The degree to which an indicator that measures a construct is reliable.	Over 95 and 100% of the outer loadings are greater than 0.7 and 0.6, respectively, which are acceptable (40). For the control design and diagnosis-report interface models, the third item in the perceived risk construct was removed for being less than 0.4.
Internal consistency reliability	A measure of the extent to which a construct's set of indicators has similar scores.	The Dillon-Goldstein metric (DG.rho) for each construct in the respective measurement models was greater than 0.7.
Convergent validity	A measure of how well the indicators that measure a construct are closely related.	The Average Variance Extracted for each of the constructs in the respective, measurement models was greater than 0.5.
Discriminant validity	A measure of the extent to which the indicators that measure a given construct are unrelated to other constructs.	The crossloading criterion for each construct was used and no indicator loaded higher on any other construct than the one it was designed to measure.

### 5.2. Analysis of Overall Structural Model

**Figure 6:**
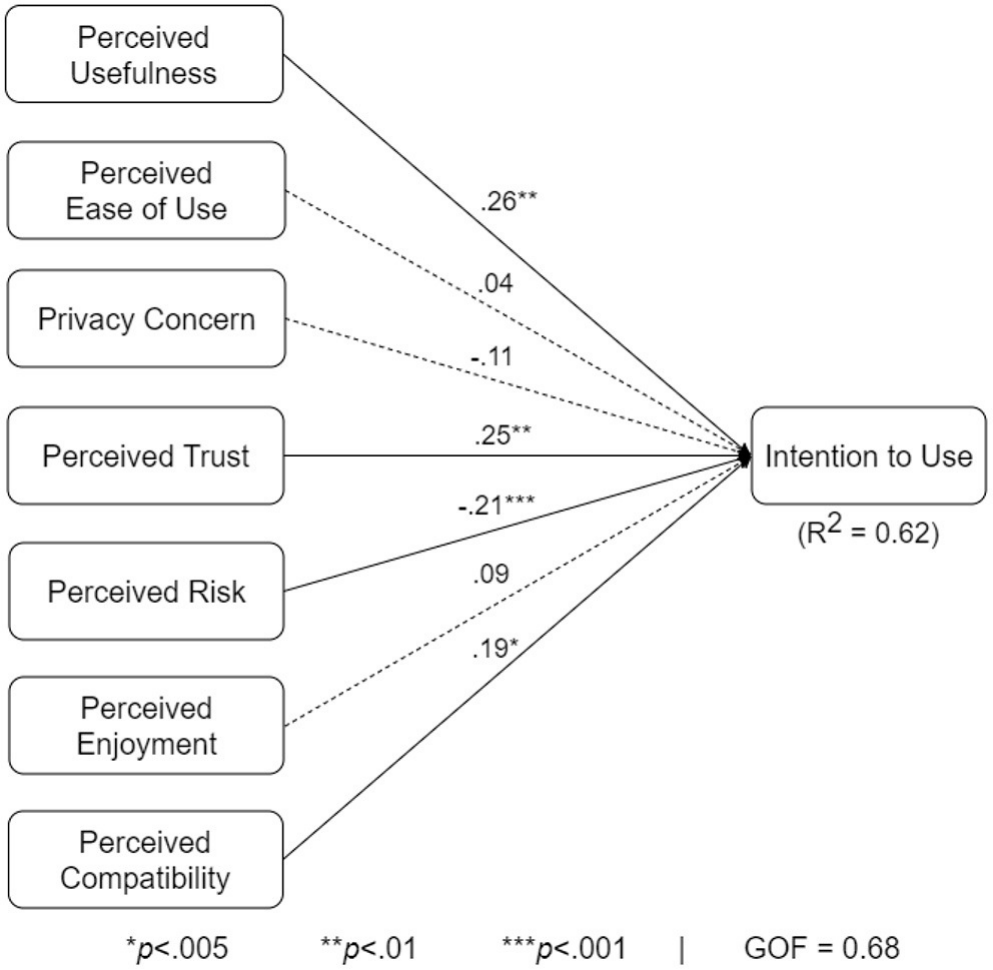
UTAUT model for the overall population.

Figure 6 shows the overall model of the intention to use the COVID Alert app. The path coefficient (β) represents the strength of the relationship between the determinants and the target construct. The coefficient of determination (*R*^2^) represents how much of the variance of intention to use the hypothesized determinants explain. Finally, the goodness of fit (GOF) indicates the extent to which the model is validated by the data. With a GOF of 68%, the overall model explains 62% of the variance of intention to use. Perceived usefulness (β = 0.26, *p* < 0.01), perceived trust (β = 0.25, *p* < 0.01), perceived risk (β = −0.21, *p* < 0.001), and perceived compatibility (β = 0.19, *p* < 0.05) are significant. However, perceived ease of use and privacy concern are not significant.

### 5.3. Multigroup Analysis of Subgroup Structural Models

**Figure 7:**
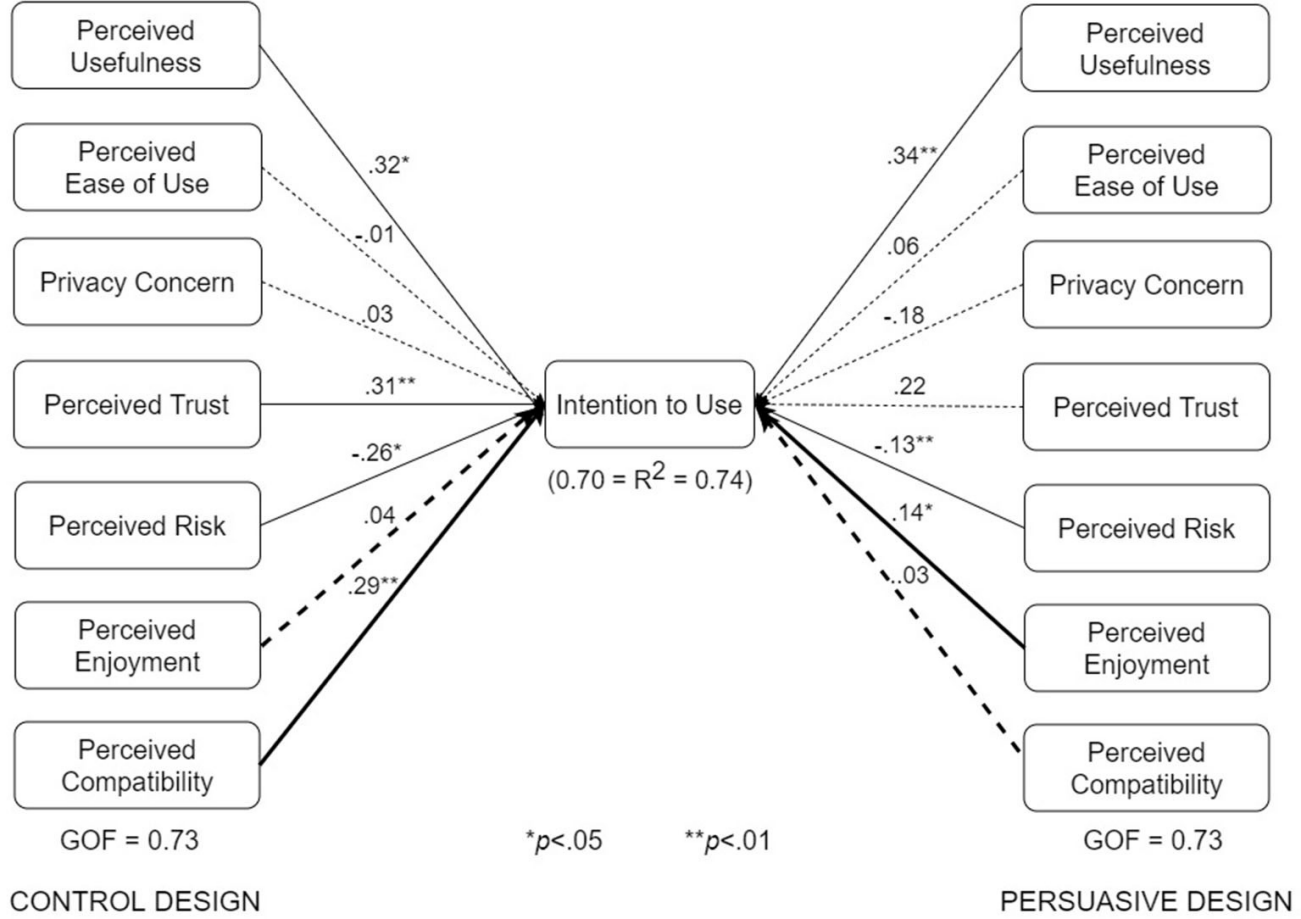
UTAUT models for control and persuasive designs (bold paths are significantly different at *p* < 0.05).

**Figure 8:**
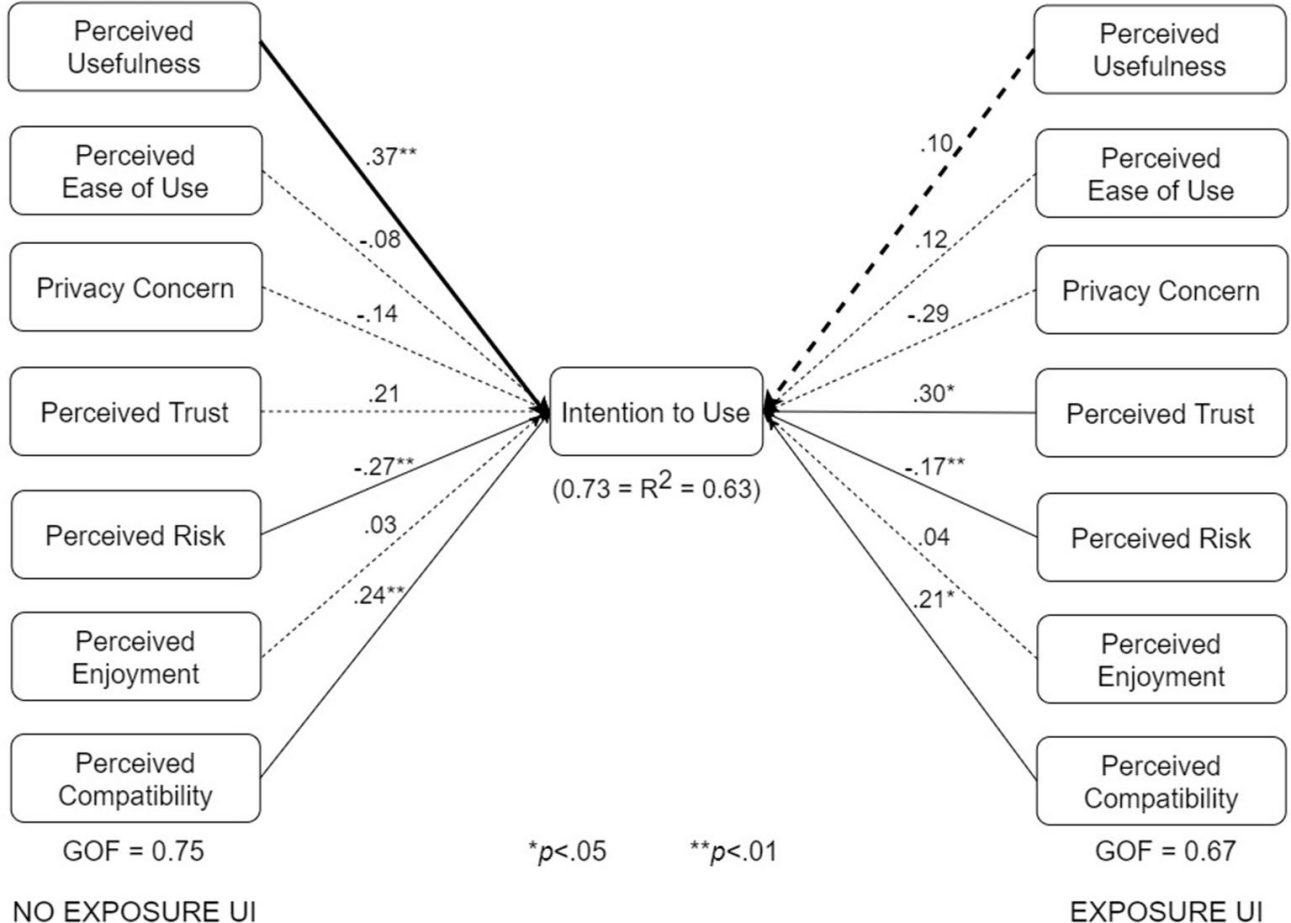
UTAUT models for no-exposure and exposure interfaces (bold paths are significantly different at *p* < 0.05).

**Figure 9:**
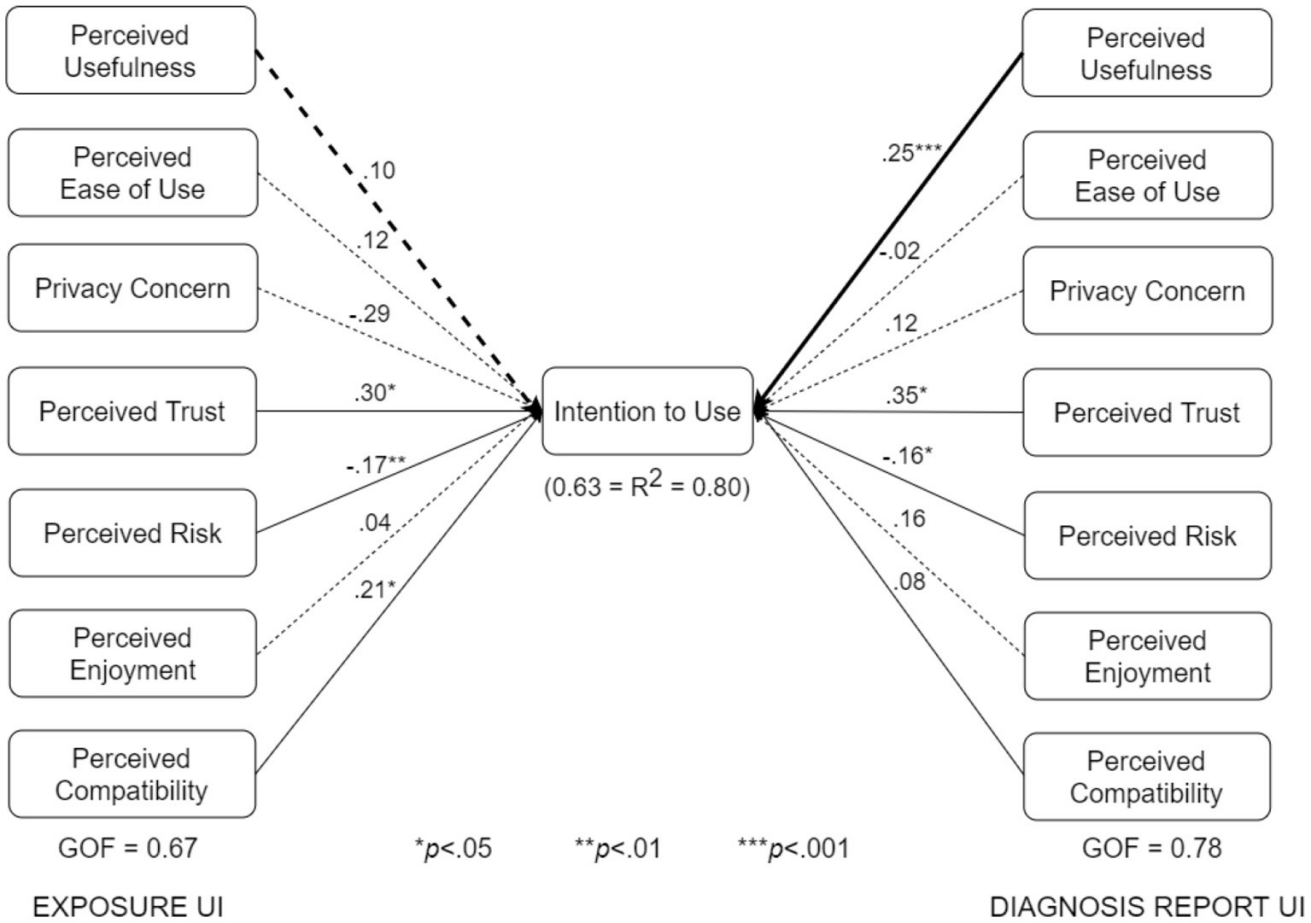
UTAUT models for exposure and diagnosis report interfaces (bold paths are significantly different at *p* < 0.05).

**Figure 10:**
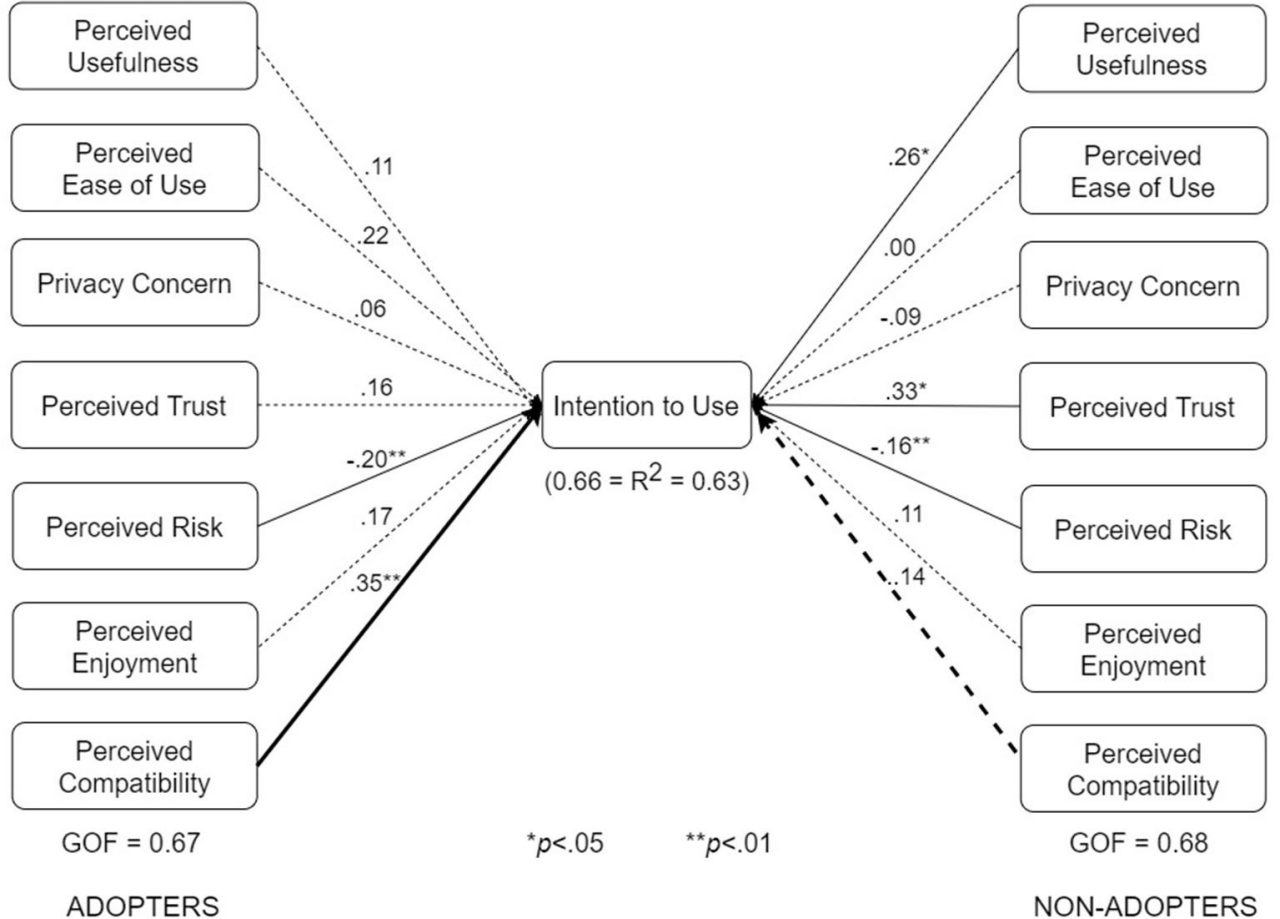
UTAUT models for adopters and non-adopters (bold paths are significantly different at *p* < 0.05).

We conducted multigroup analyses based on type of design, use case, and adoption status. The results showed that all three factors moderate one or more of the relationships in the respective pairs of submodels shown in Figures 7–10.

The various submodels were built by combining two or more subgroups in Table 3. Regarding Figure 7, the control design submodel (*n* = 101) was built by combining the corresponding no-exposure interface group (*n* = 33), exposure interface group (*n* = 36), and diagnosis-report interface group (*n* = 32). Similarly, the persuasive design submodel (*n* = 103) was built by combining the corresponding no-exposure interface group (*n* = 35), exposure interface group (*n* = 39), and diagnosis-report interface group (*n* = 29). The overall dataset used in building the structural models in Figure 7 is approximately equally split between the control design submodel (*n* = 101) and the persuasive design submodel (*n* = 103). Regarding Figure 8, the no-exposure interface submodel (*n* = 68) was built by combining the associated control design group (*n* = 33) and persuasive design group (*n* = 35). Similarly, the exposure interface submodel (*n* = 75) was built by combining the associated control design group (*n* = 36) and persuasive design group (*n* = 39). Moreover, regarding Figure 9, the diagnosis-report interface submodel (*n* = 61) was built by combining the associated control design group (*n* = 32) and persuasive design group (*n* = 29). Finally, regarding Figure 10, the adopter and non-adopter submodels were built using 82 participants (COVID Alert and ENA/CTA adopters) and 116 participants (non-adopters), respectively, as earlier discussed. It is worth noting that unlike the other comparative submodels that differ slightly based on sample size, the adopter and non-adopter submodels differ by over 30 data points. However, despite this relatively high sample-size difference, both submodels satisfy the “10-times rule-of-thumb” requirement for building a submodel: the sample size must be greater than 10 times the maximum number of links terminating in a latent variable (41). As seen in the individual path models, we have seven links terminating in the target construct (intention to use), meaning we require at least 70 data points to build each of the submodels. Apart from the diagnosis-report interface submodel (*n* = 61) and the no-exposure interface submodel (*n* = 68), every other sumodel meets the “10-times rule-of-thumb” requirement.

The multigroup analyses (Figures 7–10) show that in each pair of compared submodels, there is a significant difference between the leftside and rightside submodels regarding one or two of the seven relationships. In the submodels based on type of design (Figure 7), perceived compatibility in the control design model (β = 0.29, *p* < 0.01) is significantly different (*p* < 0.05) from that in the persuasive design model (β = 0.03, *p*>0.05). On the flip side, perceived enjoyment in the persuasive design model (β = 0.14, *p* < 0.05) is significantly different from that in the control design model (β = 0.04, *p*>0.05). Secondly, in the submodels based on use case (Figure 8), perceived usefulness in the no-exposure interface model (β = 0.37, *p* < 0.01), is significantly different from that in the exposure interface model (β = 0.10, *p*>0.05). Similarly, in Figure 9, perceived usefulness in the diagnosis-report interface model (β = 0.25, *p* < 0.001), is significantly different from that in the exposure interface model (β = 0.10, *p*>0.05). Finally, in the submodels based on adoption status (Figure 10), perceived compatibility in the no-exposure interface model (β = 0.35, *p* < 0.01), is significantly different from that in the exposure interface model (β = 0.14, *p*>0.05). Overall, the diagnosis-report interface model has the highest GOF (78%) and *R*^2^ value (80%), indicating that this subgroup is the most homogeneous among the different subsamples, into which the dataset is segmented. It is noteworthy that perceived risk has a significant relationship with intention to use in the overall model and all of the submodels, making it the most consistent significant determinant. This is not the case with the other significant constructs in the overall model (Figure 6), which are only significant in a number of the submodels. For example, perceived usefulness, although significant in the overall model, is not significant in the exposure interface submodel (β = 0.10, *p*>0.05) and the adopter submodel (β = 0.11, *p*>0.05).

### 5.4. Summary of the Results of Path Analyses

**Figure 11:**
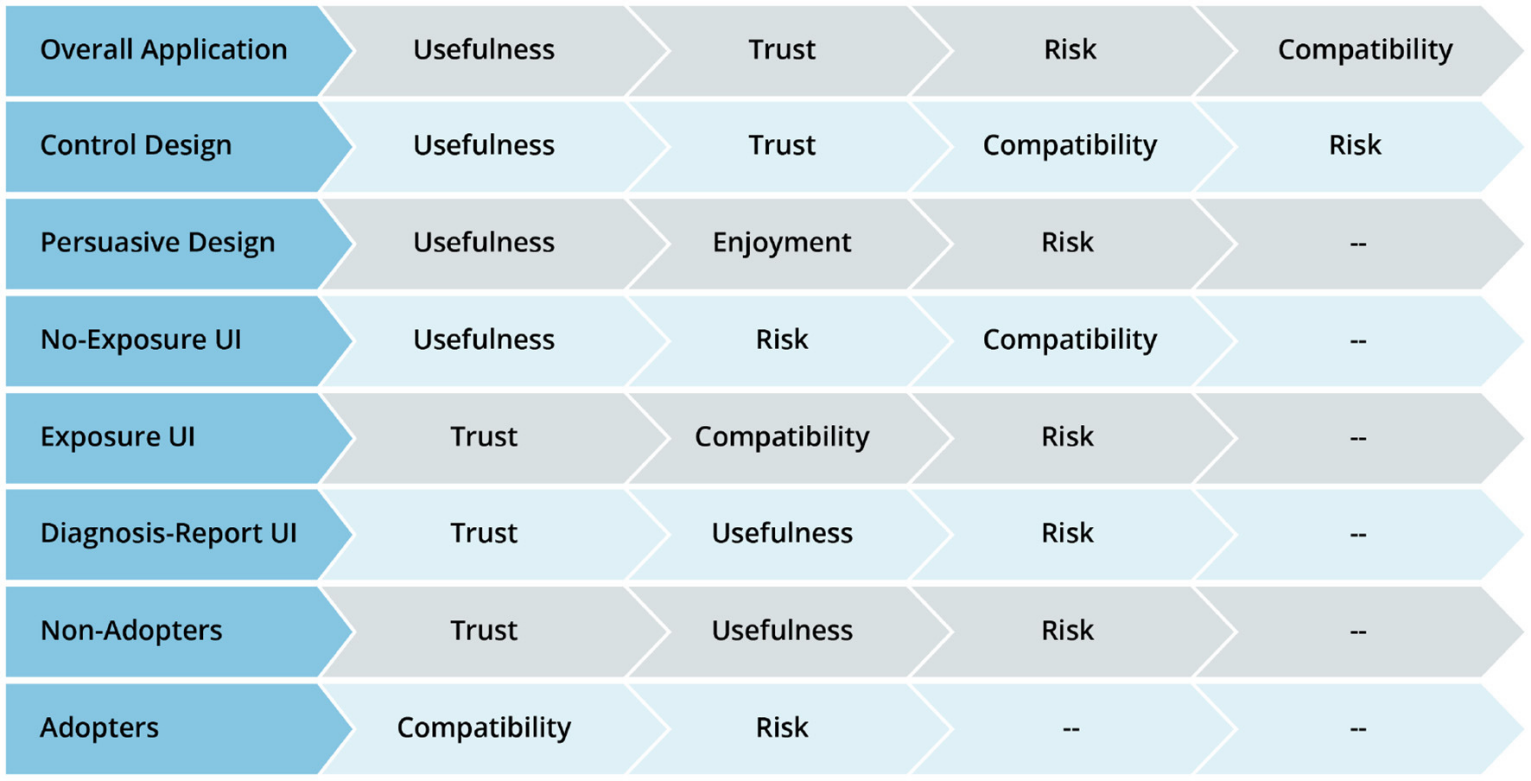
Determinants of intention to use ENA in order of strength based on path coefficient.

Table 5 summarizes the results of the structural and multigroup analyses. Perceived risk turns out to be the most consistent significant determinant of intention to use, followed by perceived usefulness, perceived trust, and perceived compatibility. Comparatively, perceived usefulness is significantly stronger in the no-exposure and diagnosis-report interface models than in the exposure interface models. Moreover, perceived enjoyment is significantly stronger in the persuasive design model than in the control design model. However, perceived compatibility is significantly stronger in the control design model than in the persuasive design model. Similarly, perceived compatibility is significantly stronger in the adopter model than in the non-adopter model. Secondly, Figure 11 summarizes the results of the structural analyses for the overall model and submodels in order of strength (magnitude) of path coefficients. Overall, perceived usefulness has the strongest effect on intention to use, followed by perceived trust, perceived compatibility and perceived risk. For example, among the eight models, perceived usefulness comes first four times (50%) and second twice (25%). Moreover, perceived trust comes first three times (33%) and second twice (25%). Although perceived risk occupies the last (third and fourth) positions more than the other determinants, it turns out to be the most consistent determinant of intention to use, as it is significant in all of the models.5

**Table 5:** Summary of main findings based on path analysis.

**Model**	**Usefulness**	**Ease of use**	**Privacy**	**Trust**	**Risk**	**Enjoyment**	**Compatibility**
Overall	✓			✓	✓		✓
Control Design	✓			✓	✓	—	✓*
Persuasive Design	✓				✓	✓*	—
Adopters					✓		✓*
Non-Adopters	✓			✓	✓		—
No-Exposure Interface	✓*				✓		✓
Exposure Interface	—			✓	✓		✓
Diagnosis-Report Interface	✓*			✓	✓		

“✓” indicates hypothesis is supported, blank cell indicates hypothesis is not supported, “—” indicates hypothesis is not supported compared with that above or below that is supported or significantly different (p < 0.05) in the multigroup analysis. “*” indicates that the path coefficient (signified by checkmark) for the subgroup is significantly stronger (p < 0.05) than its counterpart (signified by dash), with which it is compared in the multigroup analysis.

### 5.5. Discussion

We have presented the UTAUT model of the intention to use ENAs to uncover the key factors that drive adoption using the COVID Alert app as a case study and the moderating effect of type of design, use case, and adoption status. The submodels explain between 60% and 80% of the variance of intention to use, with a goodness of fit that ranges from 60% to 70%. These values indicate that the UTAUT model has a large explanatory power (42) and fits the data well to a large degree (39). In this section, we discuss the validation of the hypotheses and provide data-driven recommendations for the design of ENAs in the future, especially for the Canadian population.

### 5.6. Validation of Hypotheses

The results of the path analyses (Table 5) show that eight of the ten hypotheses are fully or partially validated. Five of the eight validated hypotheses relate to perceived risk (H5), perceived trust (H4), perceived usefulness (H1), perceived compatibility (H7), and perceived enjoyment (H6). The first two (perceived risk and perceived trust) relate to data security, while the third (perceived usefulness), fourth (perceived compatibility), and fifth (perceived enjoyment) have to do with utility, facilitating conditions, and hedonic motivation, respectively. The other three validated hypotheses have to do with persuasive design (H8), use case (H9), and adoption status (H10) moderating the strengths of some of the relationships between the determinants and intention to use.

#### 5.6.1. Data Security

Overall, users are mostly concerned about the security of their data while using ENAs. This data security concern is reflected in perceived trust and perceived risk, which have significant effects on intention to use in five and all of the eight models, respectively. For example, in the overall model, perceived risk (the belief that an ENA will violate its privacy and confidentiality norms) has a negative impact on intention to use (β = −0.21, *p* < 0.001). This effect is regarded as strong (β≥0.20, *p* < 0.05) (41). Hence, the fifth hypothesis (H5), “*The higher users perceive the design of an ENA to be risky in terms of data privacy and confidentiality, the lower will be their intention to use it*,” is validated. This finding aligns with the validation of the fourth hypothesis (H4), “*The higher users perceive the design of an ENA to be trustworthy, the higher will be their intention to use it*.” The effect of perceived trust on intention to use is strong (β = 0.25, *p* < 0.01) as well. These findings indicate that users value the security of their data. Hence, for the target population to use an ENA to curb the spread of the virus, the app has to ensure and demonstrate the security of users' privacy and personal data if it is going to collect such data.

Moreover, the app must demonstrate how users' data are going to be collected, the type, what they will be used for, whom they will be shared with, when the data will be accessed, etc. (43). For example, the ENA source code can be made public to foster trust. For instance, in the course of rolling out the Singaporean CTA, the Foreign Minister promised to make the source code freely available to developers worldwide by remarking, “*We believe that making our code available to the world will enhance trust and collaboration in dealing with a global threat that does not respect boundaries, political systems or economies*” (44). This kind of transparency has the potential of fostering public trust in the app and the various stakeholders engaged in combating the spread of the virus through contact tracing and exposure notification. The significance of perceived trust and perceived risk in the path model is in line with prior recommendations for designing ENAs/CTAs. According to privacy and technology experts, “*The success of a mobile app to contact trace Covid-19 cases depends on whether its users can trust their data will be protected*.” Our empirical findings confirm this statement made during the onset of the COVID-19 pandemic in the second quarter of 2020. Moreover, our finding replicates Kukuk's (2) finding in the context of UTAUT. The author found that perceived credibility (related to perceived trust), following behind perceived usefulness, is the second strongest determinant of intention to use a CTA.

#### 5.6.2. Application Utility

Apart from data security, users care about the utility of the app (perceived usefulness). This factor, just like perceived risk and perceived trust, has significant influence on intention to use in seven of the eight models. For example, in the overall model, the influence (β = 0.26, *p* < 0.01) is strong. Hence, the first hypothesis (H1), which has to do with the utility value of the COVID Alert app, “*The higher users perceive the design of an ENA to be useful, the higher will be their intention to use it*,” is validated. More importantly, the first hypothesis is validated in all of the submodels, except. One plausible explanation for this findings is that the adopter group takes usefulness for granted given that the participants were already using the COVID Alert app or other CTAs as of the time of completing the study. Hence, perceived usefulness tends not to matter anymore to the adopter group (Table 5). We also see the non-importance of usefulness to the adopter group reflect in other factors, such as trust, which are important to the non-adopter group. Moreover, the non-significance of perceived trust in the adopter model (Figure 10) is an indication it is no longer of importance to the adopter group as of the time of the study. In other words, the adopter group has gone past or overcome the issue of trust, which may be the first stumbling block or barrier to accepting ENAs/CTAs. Hence, trust does not matter any longer to the adopters just like usefulness and privacy concerns, all of which have no significant effect on their intention to use the COVID Alert app. However, among the non-adopter group, perceived usefulness as well as perceived trust does matter, with the former construct (β = 0.26, *p* < 0.05) and the latter construct (β = 0.33, *p* < 0.05) having a significant effect on intention to use, unlike among the adopters, where they are non-significant. Moreover, the influence of perceived usefulness tends to be significantly stronger in the no-exposure interface (β = 0.37, *p* < 0.01) and diagnosis-report interface (β = 0.25, *p* < 0.001) than the exposure interface, where it is nonsignificant (β = 0.10, *p*>0.05). One possible reason for this significant difference is that the app users who get an exposure notification (represented by those who evaluated the exposure interface) will tend to focus on the trustworthiness of the exposure information they have received. Also, they will focus on what to do next using the app, as the “Find what to do next” button in the exposure interface shows. Hence, apart from perceived risk, which is important for all of the subgroups, we see (1) perceived trustworthiness being important to the exposure-interface participants (β = 0.30, *p* < 0.05), but not to the no-exposure-interface participants (β = 0.21, *p*>0.05); and (2) facilitating conditions being important to the exposure-interface participants (β = 0.21, *p* < 0.05), but not to the diagnosis-report-interface participants (β = 0.08, *p*>0.05), who only have to simply enter their one-time key using the interface.

With that said, overall, it may not be surprising that perceived usefulness, which is significant in six of the eight models turns out to be one of the four most important factors that influence users' intention to use an ENA. This finding is consistent with existing findings in the traditional and modern domains of technology acceptance, in which perceived usefulness is found to be one of the strongest determinants of the use of an information system (10). For example, in the persuasive technology domain, Oyibo and Vassileva (29) found that perceived usefulness is the strongest determinant of intention to use a fitness app. Similarly, in the health domain, Wu et al. (45) found that perceived usefulness has the strongest influence on health professionals' intention to use a mobile health system. More recently, in the context of CTAs, Kukuk's (2) found that perceived usefulness is the second strongest determinant of intention to use in the UTAUT model. In particular, several studies such as (46) have shown that perceived usefulness has the potential to impact the actual usage of information systems.

However, with regard to utility, the second hypothesis, “*The higher users perceive the design of an ENA to be easy to use, the higher will be their intention to use it*,” is not supported by the data analysis, regardless of the app design, use case, and adoption status. One possible reason that perceived ease of use (a utility construct) does not have a significant (direct) effect on intention to use is that it is an antecedent of perceived usefulness, indicating that it can influence intention to use through perceived usefulness (10, 46). However, in this paper, we did not investigate the indirect influence of perceived ease of use and the other constructs in the model on intention to use. In future work, we will endeavor to investigate this hypothesis, in the context of ENA adoption.

#### 5.6.3. Facilitating Conditions

Apart from data security and utility of the app, users care about facilitating conditions such as familiarity with the app design. This enables users to leverage prior knowledge of similar apps in operating the ENA. The path analysis shows that facilitating condition can influence the intention to use an ENA (β = 0.19, *p* < 0.05). Hence, the seventh hypothesis (H7), “*The higher users perceive the facilitating conditions required for the effective use of ENAs, the higher will be their intention to use it*,” is validated. Moreover, this hypothesis is supported in four of the seven models (Table 5). Particularly, the relationship is stronger in the model for the control designs (β = 0.29, *p* < 0.05) than for the persuasive designs, in which it is non-significant (β = 0.03, *p*>0.05). One plausible reason for this finding is that about one third of the participants are familiar with the control design (COVID Alert app), which employs a minimalist approach. Given that they had been using the control designs prior to the completion of the survey, the participants were more familiar with its look and feel and how it worked, compared with the persuasive designs, which we presented to them for the first time. Hence, we see the participants' higher level of perceived compatibility, such as having the knowledge necessary to use the app, which, research has shown, increases perceived usefulness (35), translate into a higher intention to use the app for the control designs than for the persuasive designs. Particularly, this explanation is corroborated by the stronger relationship between perceived compatibility and intention to use the app for the adopters (β = 0.35, *p* < 0.01) than that for the non-adopters (β = 0.14, *p*>0.05). Hence, we recommend, to sustain the continued use of the ENA by adopters, especially non-adopters that became adopters, designers must promote a user-friendly design and provide users with the necessary information required to use the app effectively. For example, a help feature could be provided in the app to help users familiarize themselves with the app's functionality.

Moreover, ENAs should be designed in a way that fosters equitable access (47). For example, over two billion of the global populations still do not have a mobile phone, especially in developing countries. Even a substantial number of those that own a mobile device can only boast of a feature phone, which does not support apps. Globally, less than 50% of the world owns a smartphone. Specifically, 26.53% of people in the top 10 developed countries and 74.61% of people in the top 10 developing countries don't own a smartphone (48). As a result, designing ENAs/CTAs for non-smartphone users will facilitate the containment of the coronavirus through increased contact tracing. In this regard, Kleinman and Merkel (49) recommended that “*to ensure equitable access and to enhance the effectiveness of contact tracing, governments should provide low-cost devices to individuals without Bluetooth-enabled smartphones*.” Specifically, Nauck (50) recommended that “Bluetooth beacons” that serve the same purpose as CTAs be mass-produced and distributed to those people (e.g., children, homeless people, etc.) who do not have Android or iOS smartphones. Also recommended for contact tracing are Quick Response (QR) barcodes that can be scanned by phones and located in public spaces such as public transit bus, store and religious worship center entrances. This technology is currently being used in China as of the time of writing the paper (49).

### 5.7. Recommendations for Practice

**Figure 12:**
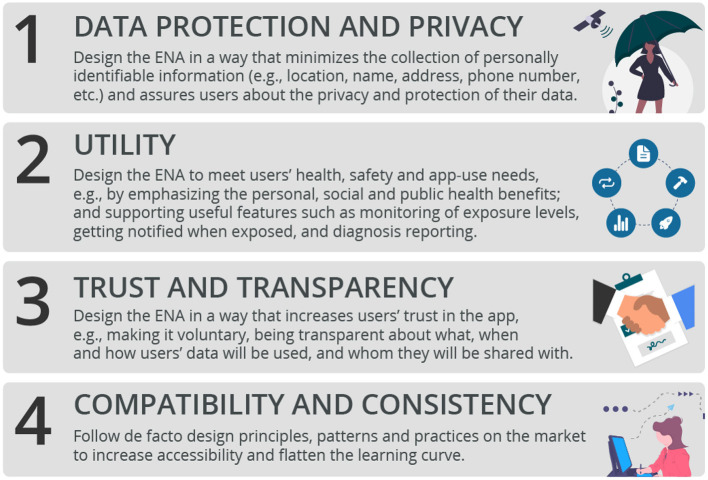
Data-driven guidelines for the design of ENAs.

Based on the significant determinants in the overall model and submodels, Figure 12 summarizes the key factors stakeholders should focus on in the design of ENAs, especially for the Canadian population (2, 51, 52). First and foremost, ENA stakeholders should focus on addressing user data protection and privacy. For example, they should design the ENA in a way that minimizes the collection of personally identifiable information (e.g., location, name, address, mobile number, etc.). Moreover, they should design the app in a way that assures users about the privacy, confidentiality and protection of their data. This has the potential of reducing the perceived risk associated with the use of ENAs by the Canadian population.

Secondly, designers should focus on making the app very useful to the users. One way to disseminate the perceived usefulness of an ENA is to emphasize its health and safety utility to the individual, community and public at large. For example, while announcing the roll-out of the Singaporean ENA, “TraceTogether,” the government emphasized the health and safety utility of the app: “*Together we can make our world safer for everyone*” (17, 44). Moreover, as described by (15), persuasive features such as self-monitoring and social learning can be incorporated into the interface design of ENAs to increase their uptake and make them more effective. For example, in the submodels for the two types of design (Figure 7), there is a significant relationship between perceived enjoyment and intention to use for the persuasive design, but none for the control design. According to (53), users do not only want to use an app for its utilitarian purpose, they want to desire and enjoy using it as well. The persuasive version of the ENA has the potential to realize this. As shown in the persuasive design model (Figures 7, 10), the higher users' perception of enjoyment of the ENA is, the more likely they are to adopt it.

Thirdly, designers and stakeholders should focus on engendering trust in the design of ENAs. Specifically, for people yet to adopt ENAs (whom are the focus of a study on technology acceptance), as shown in Figure 10, fostering trust should be the most important issue designers should be concerned with. For example, the ENA should be designed in a way that increases users' trust, which includes making it voluntary and being transparent about what, when and how users' data will be collected and used, and whom they will be shared with eventually.

Fourthly, designers and stakeholders should focus on data protection, privacy and confidentiality by showing and/or demonstrating to potential users what data will be collected, at what time, how it will be used, who will have access to it and when, etc. Moreover, for adopters, designers should focus primarily on making the app easier to use by leveraging de facto design principles, which the users are familiar with. This recommendation aligns with Nielsen's (54) fourth heuristic for usability, “Consistency and Standards,” which discourages reinventing the wheel. Consistency can be compared to Moore and Benbasat's “Compatibility” construct, which is defined as “*the degree to which an innovation is perceived as being consistent with the existing values, needs, and past experiences of potential adopters*” (14). Having so much experience with various interfaces has made the human brain and mind create certain patterns and mental models, which make users look for certain elements and functionality in certain places in an application (55). Fostering interface design consistency and standards has the potential of improving the overall user experience and keeping adopters interested in using the app for a very long time (56).

### 5.8. Limitations and Future Work

Our study has a number of limitations. The first limitation is that our findings are based on users' perceptions. This may limit the generalization of our findings to the real-world context of ENA use. The second limitation of our study is that our sample size is relatively small (*n* = 204) compared to the larger Canadian population of over 35 million. This warrants further studies of a larger sample size to investigate how well our findings are able to generalize to the large population. The third limitation of our study is that we did not investigate the interrelationships among the proposed factors of ENA adoption. It may turn out that while some of the hypothesized factors (e.g., privacy concern) do not have a direct relationship with intention to use, they may indirectly. Future analyses will help us uncover this hypothesis. The fourth limitation of the study is that our path analysis did not consider the moderating effect of demographic factors, such as country of origin, culture, age, education level, social status, etc. Hence, more research needs to be done in this area. Particularly, in the near future, we hope to extend our study to the American and Nigerian populations, which are similar to and different from the Canadian population in terms of culture and socio-economic development, respectively. This will help us to uncover how the current findings generalize to a country with a similar and different culture. The fifth limitation is that, in defining non-adopters, we did not distinguish those who used the app (occasionally or regularly) from those who just downloaded it from the app stores and never used it. Future work can address this limitation by asking participants about the extent of adoption. The sixth limitation is regarding the utility of ENAs in fighting the pandemic. We acknowledge that while ENAs can help and offer some benefit in curbing the spread of COVID-19, they may not be the ultimate solution or silver bullet to returning the globe to normalcy. This is due to limitations in technology (e.g., lack of interoperability), unavailability in some countries (e.g., developing countries in Africa), limitation to smartphones, unwillingness by some individuals to adopt the technology, and the nature of how infections work and spread (57).

### 5.9. Contributions

This paper makes a number of contributions to the existing literature on technology acceptance of CTAs/ENAs. It is the first to investigate the relationships between the UTAUT exogenous constructs (related to utility, security, hedonism, and compatibility) and endogenous construct (intention to use) considering the three main use cases of ENAs: no-exposure status, exposure status, and diagnosis reporting. The second contribution is that the paper is the first to investigate the technology acceptance model for the Canadian population by using Canada's official ENA (COVID Alert) as a case study. Finally, the paper is the first to consider the moderating effect of type of design, use case, and adoption status by showing empirically how the UTAUT models for the various subgroups significantly differ.

## 6. Conclusion

In this paper, we presented the key factors that determine the acceptance of an ENA on the market using the Canadian COVID Alert app as a case study. The results of our path modeling show that perceived risk, perceived trust, perceived usefulness and perceived compatibility are significant determinants of the intention to use an ENA. Moreover, our multigroup analyses showed that the relationships between these constructs and intention to use are moderated by type of design, use case, and adoption status. The relationship between perceived enjoyment and intention to use is significant for the persuasive design, but non-significant for the control design. On the other hand, the relationship between perceived compatibility and intention to use is significant for the control designs but non-significant for the persuasive designs. Similarly, the relationship between perceived compatibility and intention to use is significant for the adopters, but non-significant for the non-adopters. Finally, the relationship between perceived usefulness and intention to use is significant for the no-exposure and diagnosis-report interfaces, but non-significant for the exposure interface. Based on our findings, we provided design recommendations, which stakeholders can adopt in creating more effective ENAs/CTAs in the future. In the context of path modeling, our study is the first to explore the moderating effect of important factors such as type of design, use case, and adoption status on the acceptance of ENAs using COVID Alert (an actual app) as a case study. This holds the potential of tailoring ENAs to adopters and non-adopters, for example, by focusing on perceived trust and usefulness among the later group.

## Data Availability Statement

The datasets presented in this article are not readily available because ethics requirements do not allow releasing the data without a new behavior ethics approval. Requests to access the datasets should be directed to Plinio Pelegrini Morita at plinio.morita@uwaterloo.ca.

## Ethics Statement

The study involving human participants were reviewed and approved by the Research Ethics Board of the University of Waterloo. The participants provided their written informed consent prior to participating in the study.

## Author Contributions

PM funded the study with his grant. KO recruited the participants, cleaned and analyzed the data, and wrote the paper. Both authors designed the study, contributed to the article, and approved the submitted version.

## Conflict of Interest

The authors declare that the research was conducted in the absence of any commercial or financial relationships that could be construed as a potential conflict of interest.

## Publisher's Note

All claims expressed in this article are solely those of the authors and do not necessarily represent those of their affiliated organizations, or those of the publisher, the editors and the reviewers. Any product that may be evaluated in this article, or claim that may be made by its manufacturer, is not guaranteed or endorsed by the publisher.

## Appendix - Description of Survey Administration

In our study, we administered three key functional Interfaces (in the form of screenshots) to participants in the persuasive and control groups. Table A1 shows the interfaces (non-interactive mock-ups) presented to each of the six groups in the study. In the control groups, we presented the three control versions (C1, C2, and C3) and asked the study participants to answer the questions presented in Table 2 regarding each of the versions. Similarly, in the persuasive groups, we presented one of the persuasive versions (P1, P2, and P3) alongside two of the control versions and asked the study participants to answer the questions presented in Table 2 regarding each of the persuasive versions. For example, for the persuasive no-exposure interface group, we presented the persuasive version of the no-exposure interface (P1) and the control versions of the exposure interface (C2) and diagnosis-report interface (C3). We took this approach (presenting all three functional interfaces simultaneously) so that each group would have an overall view of the COVID Alert app and how it functions.

The following description was provided about each of the three interfaces (see Figures 2, 3) presented to each of the six groups of participants.

Below is the Government of Canada's Covid Alert app. The app can let you and other users know of possible COVID-19 exposures before any symptoms appear. We would like you to take some time (at least 2 min) to study the three interfaces.

**Screen 1** is the “No Exposure Notification” interface, which lets you know that you have not been exposed to COVID-19 by being close to an infected person.

**Screen 2** is the “Exposure Notification” interface, which lets you know that you may have been exposed to COVID-19 and what to do next.

**Screen 3** is the “Diagnosis Key Entry” interface, which allows you to enter your one-time key given to you by the public health authority if diagnosed with COVID-19.

When the key (in Screen 3) is entered into the app, other users, who may have come into close contact with the user who entered the key, are sent a notification (Screen B) and provided guidance on what to do next (e.g., self-isolate or go test for COVID-19 in the event of having symptoms).

The app uses strong measures to protect any data it collects, and does not track a user's location or collect personally identifiable information such as name, contacts, address or health information.

In the study, we asked participants in each group to focus on the functional interface assigned to the group as follows:

**Groups 1A and 2A**. Assuming you were using the Covid Alert app and you got the following exposure notification [image of no-exposure interface] on your mobile phone, kindly answer the following questions based on the information on the screen.

**Groups 1B and 2B**. Assuming you were using the Covid Alert app and you got the following exposure notification [image of exposure interface] on your mobile phone, kindly answer the following questions based on the information on the screen.

**Groups 1C and 2C**. Assuming you were using the Covid app and were diagnosed with COVID-19 by public health and given a one-time key to be entered into the Covid Alert app as shown below [image of diagnosis-report interface], kindly answer the following questions based on the information on the screen.

**Table A1:** Three functional interfaces in each of the two app designs presented to six groups of study participants.

	**Group**	**A**	**B**	**C**
**Control design**	1	**C1**C2C3	C1**C2**C3	C1C2**C3**
**Persuasive design**	2	**P1**C2C3	C1**P2**C3	C1C2**P3**

*The bolded interface (e.g., “P2”) is the interface of interest for the respective groups. The other (unbolded) interfaces were presented alongside the interface of interest so that participants would have an overview of the three key use cases of the COVID Alert app*.
